# High expression of *dedicator of cytokinesis 1* (*DOCK1*) confers poor prognosis in acute myeloid leukemia

**DOI:** 10.18632/oncotarget.19706

**Published:** 2017-07-31

**Authors:** Sze-Hwei Lee, Yu-Chiao Chiu, Yi-Hung Li, Chien-Chin Lin, Hsin-An Hou, Wen-Chien Chou, Hwei-Fang Tien

**Affiliations:** ^1^ Division of Hematology, Department of Internal Medicine, National Taiwan University Hospital, Taipei, Taiwan; ^2^ Graduate Institute of Biomedical Electronics and Bioinformatics, National Taiwan University, Taipei, Taiwan; ^3^ Department of Laboratory Medicine, National Taiwan University Hospital, Taipei, Taiwan

**Keywords:** DOCK1, acute myeloid leukemia, prognosis, stemness, cell migration

## Abstract

*DOCK* family genes encode evolutionarily conserved guanine nucleotide exchange factors for Rho GTPase involving multiple biological functions. Yet the patterns and prognostic significance of their expression in acute myeloid leukemia (AML) remain unexplored. Here we analyzed the expression patterns of 11 *DOCK* family genes in AML cells based on the array data of 347 patients from our cohort and several other published datasets. We further focused on the implications of the expression of *DOCK1* since it was the only one in DOCK family to be associated with survival. Physiological functions and biological pathways associated with *DOCK1* were identified using bioinformatics approaches. With a median follow up of 57 months, higher *DOCK1* expression was associated with shorter disease free and overall survival. The finding could be validated by two independent cohorts. Multivariate analysis showed higher *DOCK1* expression as a strong independent unfavorable prognostic factor. Higher *DOCK1* expression was closely associated with older age, higher platelet and peripheral blast counts, intermediate-risk cytogenetics, *FLT3*-ITD, *MLL*-PTD and mutations in *PTPN11*, *NPM1*, *RUNX1*, *ASXL1* and *DNMT3A*. Functional enrichment analysis suggested the association of *DOCK1* overexpression with several key physiological pathways including cell proliferation, motility, and chemotaxis. Therefore, we suggested that AML with higher *DOCK1* expression showed characteristic clinical and biological features. *DOCK1* expression is an important prognostic marker and a potential therapeutic target for the treatment of AML. Studies in large prospective cohorts are necessary to confirm our findings. Further mechanistic studies to delineate the role of *DOCK1* in the leukemogenesis are warranted.

## INTRODUCTION

The Dedicator of cytokinesis (DOCK) family protein is a novel class of guanine nucleotide exchange factors (GEF) for Rac GTPases of the Rho family, [1] and is known to involved in the regulation of actin cytoskeleton, cell motility, [2] as well as cell cycle, survival, gene expression, and tumorigenesis [1]. There are 11 DOCK proteins (DOCK1 to DOCK11) in mammals, which are further classified into four subgroups (denoted as A to D) according to the sequence similarity and domain organization. The proteins in DOCK-A and B subgroups contain a terminal domain that allows binding of the adaptor protein ELMO (Engulfment and Motility) to promote efficient GEF activity [2]. Knocking down *ELMO1* impairs long-term expansion of leukemic cell lines and depletion of *ELMO1* in human CD34^+^ cells inhibit proliferation, possibly through the inhibition of ELMO-Rac axis [3]. The DOCK proteins are involved in several diseases including cancers, and disorders in the immune and central nervous systems [1]. Among them, DOCK2 plays a pivotal role in lymphocyte migration and activation as well as differentiation of T cells. A recent study identified DOCK2 as one of the FLT3 interacting protein [4]. DOCK4 participates in erythroid maturation [5]. The only known function of DOCK5 so far is the connection with osteoclasts [6]. Deletion of DOCK8 in human results in a combined immune deficiency syndrome [7]. Deregulation of the remaining DOCK proteins may contribute to neurodegenerative diseases and central nervous system defects [8]. As a prototype member in DOCK family, DOCK1 is involved in migration and invasion of various cancer cells including breast cancer, ovarian cancer and glioblastoma multiforme [9–11]. Despite the protean functions of DOCK proteins in cancers and hematopoiesis, their roles in acute myeloid leukemia (AML) have not been explored. In this study, we first screened the expression levels of *DOCK* family genes in a cohort of AML patients. The members with at least modest expression in AML were selected to test their prognostic significance in ours and the other two independent cohorts. We found that expression level of *DOCK1* but not the other members harbored prominent prognostic significance in both ours and other independent cohorts of AML patients. Higher expression of *DOCK1* served as an independent unfavorable prognostic marker. The prediction power of this single gene expression even outperformed several published gene signatures in AML prognostication. Bioinformatics analyses were performed to identify potential pathway and function of *DOCK1* in AML.

## RESULTS

### Expression of *DOCK* family members in AML

**Figure 1:**
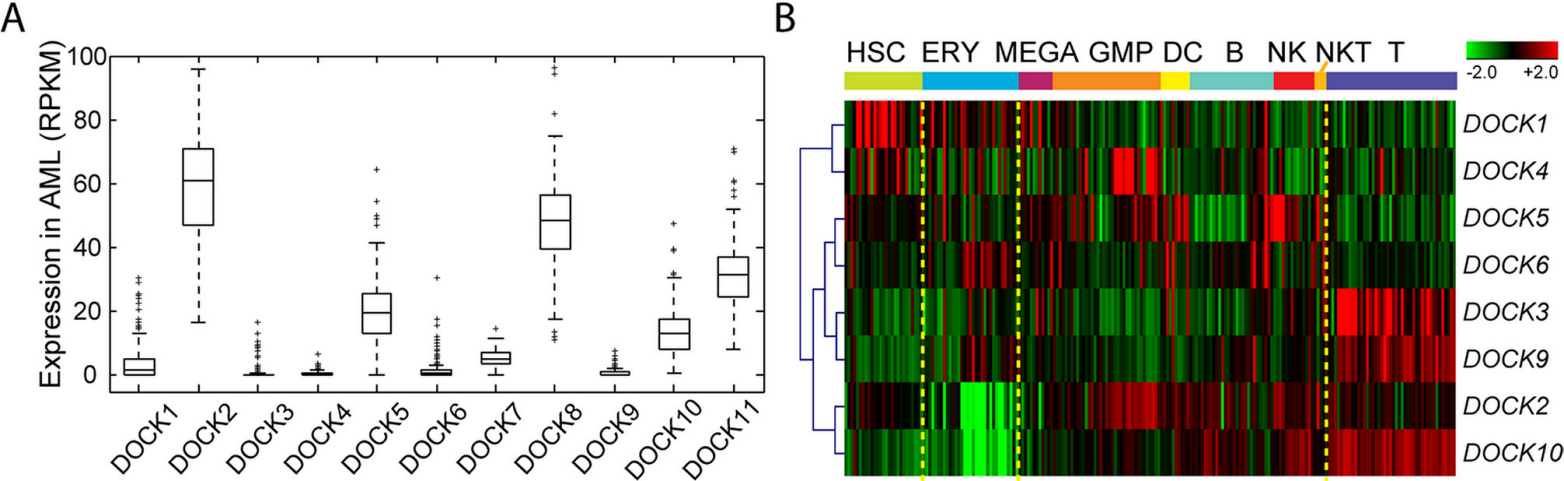
Expression profile of DOCK family genes in AML and normal samples **(A)** Expression levels of DOCK family genes in AML. Samples of 179 AML patients were profiled with Illumina RNA-Seq by TCGA. RPKM, reads per kilobase of transcript per million mapped reads. **(B)** Heatmap of DOCK genes across hematopoietic cell states in GSE24759 (n=211). Expression levels were z-transformed in each probe, and multiple probes representing the same gene were averaged. Genes were clustered using hierarchical clustering. hematopoietic stem cells (HSC), erythroid cells (ERY), megakaryocytes (MEGA), granulocyte/monocyte progenitors (GMP), dendritic cells (DC), B cells (B), natural killer cells (NK), natural killer T cells (NKT), and T cells (T).

To obtain a comprehensive expression landscape of *DOCK* family genes in hematopoietic system, we profiled gene expression microarrays of the 347 AML samples (NTUH cohort) and reprocessed several public expression datasets of AML and/or normal samples. In the TCGA RNA-Seq data of AML patients (n=179), expression levels of *DOCK* genes were quite diverse (Figure 1A). *DOCK2* and *DOCK8* were among the top 10% of genome-wide genes (median RPKM, 60.8 and 50.3), while *DOCK3*, *DOCK4*, *DOCK6*, and *DOCK9* seemed silent (all median RPKM values<2), and *DOCK5*, *DOCK10*, and *DOCK11* showed modest expression. *DOCK1* had low but measurable expression levels among the AML patients. To investigate the involvement of *DOCK* genes in hematopoiesis, we analyzed another microarray dataset of samples across various hematopoietic cell stages (GSE24759, n=211). *DOCK1* seemed to be exclusively abundant in hematopoietic stem cells (HSC) (average *z*-value=1.17; one-sample *t*-test p<0.001; Figure 1B). In erythroid cells, expression levels of *DOCK2* and *DOCK10* were especially low (average *z*, −1.53 and −1.36; both p<0.001). *DOCK3*, *DOCK9*, and *DOCK10* formed a cluster of high expression in T cells (all p<0.001; Figure 1B).

### The prognostic significance of gene expression of *DOCK* family members in AML

**Figure 2:**
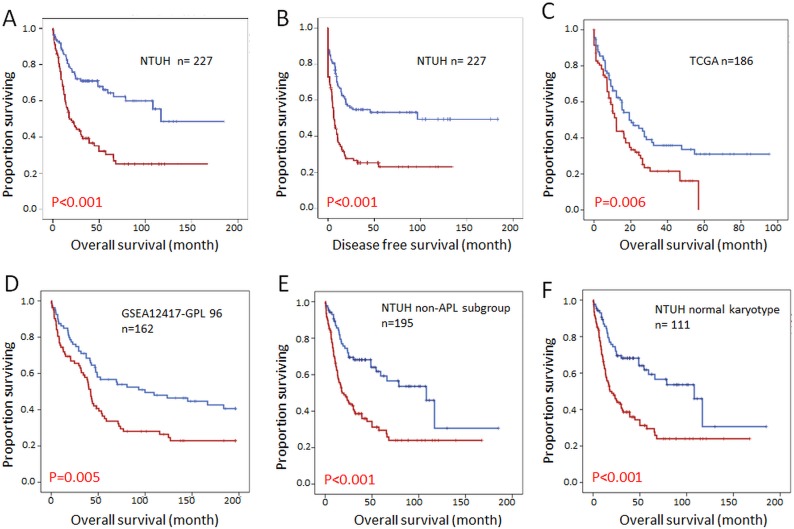
Kaplan Meier survival curves for AML patients stratified by *DOCK1* expression levels **(A)** Overall survival in the NTUH cohort. **(B)** Disease free survival in the NTUH cohort. **(C)** Overall survival in the TCGA cohort. **(D)** Overall survival in the GSEA12417-GPL96 cohort. **(E)** Overall survival in non-APL patients from the NTUH cohort. **(F)** Overall survival in AML patients with normal karyotype from the NTUH cohort. Red line: higher DOCK1 expression; blue line: lower DOCK1 expression.

Among the 347 AML patients in the NTUH cohort, 227 who received standard chemotherapy were dichotomized into two groups by the median expression levels of individual *DOCK* members according to the array data. With a median follow up of 57 months, we observed that a higher *DOCK1* expression level was associated with worse OS (median 20 vs. 116.8 months, p<0.001) and DFS (median 11.0 vs. 101.7months, p<0.001) (Figure 2A and 2B). Convincingly, these observations could be validated by two independent cohorts TCGA (N=186) and GSE12417 (N=162) (Figure 2C and 2D). The similar prognostic impact of *DOCK1* expression was also present in the patients with AML other than acute promyelocytic leukemia (Figure 2E) as well as cytogenetically normal AML (Figure 2F) in our cohort. Overall, these data confirmed that *DOCK1* expression levels harbored a significant impact on survival in AML patients. On the contrary, higher expression of *DOCK2* was associated with a favorable outcome in our patients (median OS 68.0 months vs. 24.9 months, p=0.020), however, this result was not able to be validated by TCGA or GSE12417 cohort (Supplementary Figure 1). The expression of other *DOCK* genes failed to demonstrate impacts on survival in our AML patients. We thus focused on *DOCK1* gene expression in subsequent analyses.

For the 227 patients who received standard chemotherapy, 61% of those with higher *DOCK1* levels achieved complete remission (CR), compared to 84% in the lower expression group (p<0.001, Table 1). Univariate Cox analysis revealed that higher *DOCK1* expression (p<0.001), unfavorable karyotype (p=0.001), older age (p=0.003), higher WBC count (p=0.015), CD 34 expression (p=0.018), *FLT3*-ITD (p= 0.001), *MLL*-PTD (p=0.001), and mutations in *TP53* (p<0.001), *RUNX1* (p=0.040) and *WT1* (p=0.020) were all associated with shorter OS (Supplementary Table 1) while similar parameters, except *WT1* and *DNMT3A* mutations, adversely affected DFS (Supplementary Table 2). Presence of *CEBPA* double mutations correlated with better OS and DFS. Multivariate analysis confirmed higher *DOCK1* expression, along with older age, higher WBC count, unfavorable karyotype, absence of *CEBPA* double mutations and *TP53* mutation, as an independent unfavorable prognostic factor for both overall survival and disease free survival, p=0.005 (Table 2) and p<0.001 (Supplementary Table 3) respectively.

**Table 1:** Comparison of clinical manifestations between AML patients with higher and lower BM *DOCK1* expression

Variables	Total (n=347)	*DOCK1* expression		*P* value
		Higher (n=174)	Lower (n=173)	
**Sex**^†^				0.253
Male	196	93	103	
Female	151	81	70	
**Age (year)**^‡^		62 (15-89)	53 (16-91)	0.010
**Lab data**				
WBC (/μL)^‡^		24415 (890-423000)	18770 (380-417500)	0.188
Hemoglobin (g/dL)^‡^		8.1 (3.3-14.0)	8.1 (3.7-16.2)	0.441
Platelet (×1,000/μL)^‡^		60 (7-655)	39 (2-493)	<0.001
Blast (/μL)^‡^		12325 (0-345964)	5080 (0-369070)	0.005
LDH (U/L)^‡^		897 (202-9097)	931 (242-13130)	0.412
**FAB**^†^		174	173	<0.001
M0	9	4	2	0.414
M1	67	35	32	0.703
M2	109	54	55	0.879
M3	28	1	27	<0.001
M4	103	62	41	0.015
M5	20	11	9	0.655
M6	8	4	4	0.993
Undetermined	6	3	3	0.994
**Induction response**^†^	227	114	113	<0.001
CR^†^	165 (72.7%)	70 (61.4%)	95 (84.1%)	<0.001
PR+ refractory^†^	47 (20.7%)	34 (29.8%)	13 (11.5%)	<0.001
Induction death^†^	15 (6.6%)	10 (8.8%)	5 (4.4%)	0.187
**Relapse**^†^	72 (31.7%)	40 (35.1%)	32 (28.3%)	0.273

^†^Number of patients (%).

‡Median (range).

Abbreviations: WBC, white blood cell count; FAB, French-American-British classification; CR, complete remission; PR, partial remission.

**Table 2:** Multivariate analysis (Cox regression) on the overall survival*

Variables	Overall survival			
	HR	95% CI		*P* value
		Lower	Upper	
**Total cohort (n=227)**				
Age	1.014	1.001	1.027	0.036
WBC	1.000	1.000	1.000	0.036
Unfavorable cytogenetics	2.672	1.484	4.809	0.001
*FLT3*-ITD	1.320	0.840	2.073	0.229
*CEBPA^double mutation^*	0.331	0.131	0.837	0.019
*RUNX1* mutation	1.279	0.718	2.275	0.403
*MLL*-PTD	1.636	0.729	3.620	0.225
*WT1* mutation	1.827	1.022	3.267	0.042
*TP53* mutation	3.838	1.416	10.402	0.008
***DOCK1*** **higher expression**	**1.501**	**1.127**	**1.998**	**0.005**

*The model was generated from a stepwise Cox regression model that included age, WBC, unfavorable cytogenetics, gene mutations of *FLT3*, *WT1*, *CEBPA*, *RUNX1*, *MLL*, *TP53* and expression level of *DOCK1*.

Abbreviations: HR, hazard ratio; CI, confidence interval; WBC, white blood cell count.

### Association of expression levels of *DOCK1* with clinical and biological parameters

Patient with higher *DOCK1* expression were older (p=0.010) and had higher platelet (p<0.001) and peripheral blast counts (p=0.005) at diagnosis than the lower expression group (Table 1). In addition, higher *DOCK1* expression level was closely associated with intermediate-risk cytogenetics (p=0.003) and normal karyotype (p=0.021), but inversely correlated with favorable karyotype, including t(8;21) and t(15:17) (all p<0.001) (Supplementary Table 4). Moreover, mutations in *CEBPA*, *KIT*, and *IDH2* occurred less often whilst *FLT3-ITD* and mutations in *PTPN11, MLL, NPM-1, RUNX1, ASXL1* and *DNMT3A* appeared more frequently in patients with higher *DOCK1* expression than in those with lower expression (Supplementary Table 5).

### Comparisons of prognostic significance of *DOCK1* expression with published prognostic gene signatures

To further evaluate the potential of *DOCK1* expression as a prognostic marker for AML, we compared the survival impact of *DOCK1* expression with other published gene expression-based prognostic signatures. We performed pairwise multivariate Cox analysis of *DOCK1* expression with each of the published 3-gene, [12] 7-gene, [13] 11-gene, [14] and 24-gene predictors [15]. Notably, *DOCK1* expression remained a prognostic factor independent to all these composite signatures (with multivariate Cox p<0.05) and achieved even higher significance in most of the comparison settings (10 out of 12; Table 3). Considering that these signatures were originally developed by diverse study designs in multiple AML cohorts, our data suggested *DOCK1* expression as a simple, powerful, and widely applicable prognostic marker.

**Table 3:** Comparisons of *DOCK1* expression to published prognostic gene signatures

Predictor	NTUH (n=227)	TCGA (n=186)	GSE12417 (n=162)
*DOCK1*	<0.001* (1.50;1.27-1.76)**	0.001 (1.73;1.26-2.37)	0.001 (1.34;1.12-1.60)
3-gene score (Wilop et al.)	0.010 (1.34;1.07-1.68)	0.003 (1.37;1.11-1.69)	0.194 (1.15;0.93-1.42)
*DOCK1*	<0.001 (1.43;1.20-1.70)	0.003 (1.66;1.19-2.31)	0.008 (1.28;1.07-1.55)
7-gene score (Marcucci et al.)	0.003 (1.19;1.06-1.34)	0.092 (1.10;0.98-1.22)	0.031 (1.19;1.02-1.38)
*DOCK1*	0.001 (1.39;1.15-1.67)	0.005 (1.68;1.17-2.41)	0.146 (1.17;0.95-1.44)
11-gene score (Chuang et al.)	0.007 (1.05;1.01-1.09)	0.337 (1.02;0.98-1.07)	0.004 (1.06;1.02-1.11)
*DOCK1*	0.004 (1.36;1.11-1.68)	0.034 (1.43;1.03-2.00)	0.023 (1.32;1.04-1.68)
24-gene score (Li et al.)	0.029 (1.07;1.01-1.13)	<0.001 (1.11;1.05-1.18)	0.493 (1.02;0.97-1.07)

*Pairwise multivariate *P*-value (**hazard ratio; 95% confidence interval of hazard ratio).

### Functional enrichment analysis of *DOCK1*

**Figure 3:**
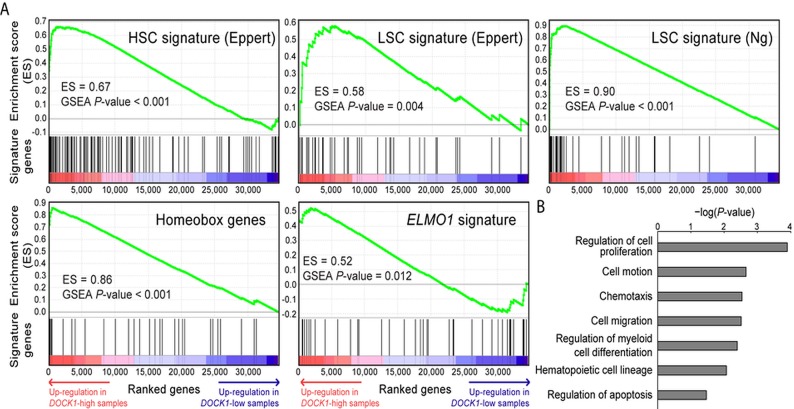
Functional enrichment analysis of *DOCK1* in AML **(A)** GSEA enrichment plots. GSEA sorted genome-wide genes by the significance of differential expression between patients with higher and lower *DOCK1* expressions (denoted by red and indigo arrows) in the NTUH cohort. Each set of genes was tested for an enrichment (measured by an enrichment score and permutation-based p-value) at either side of the list. Four gene sets were analyzed, including gene signatures of HSC and LSC, described previously by Eppert et al [16] and Ng et al [17], respectively and our manually curated *ELMO1*-interacting genes and Homeobox genes. All of these gene sets showed significant enrichment in samples with *DOCK1* up regulation. **(B)** Functions and pathways of *DOCK1* in the NTUH cohort. We used the DAVID web tool to analyze associated functions and pathways of 321 probes differentially expressed between *DOCK1*-high and -low patients. DAVID-generated modified Fisher's exact test p-values of selected terms are represented in log-10 scale.

We conducted functional enrichment analysis to gain biological insights into the underlying mechanisms of unfavorable prognosis related to *DOCK1* over-expression. The up-regulation of *DOCK1* in HSC cells (Figure 1B) implied its role in stem cell biology in AML. To test this hypothesis, we curated two published stem cell gene signatures [16, 17]. GSEA analysis showed concordant up-regulation of HSC and leukemic stem cell (LSC)-associated genes (Supplementary Tables 6 and 7) in patients with higher *DOCK1* expression (GSEA p<0.001 and p=0.004, respectively; Figure 3A). Furthermore, GSEA revealed a concordant enrichment of homeobox genes (Supplementary Table 8, p<0.001; Figure 3A), indicating the association of *DOCK1* in stem cell functions as homeobox genes are well-known central player in determining stem cell fate [18].

DOCK1 is known to interact physically with ELMO1, a mediator of chemotaxis in AML, with an implication in cell migration [2, 3]. We reasoned that the adverse effect of higher *DOCK1* expression in prognosis might be mediated by this axis. To verify this, we manually curated a signature of *ELMO1*-interacting genes (Supplementary Table 9) to measure its activity and ran another GSEA analysis. Indeed, the *ELMO1* signature was positively associated with *DOCK1* overexpression (p=0.012; Figure 3A). DAVID analysis confirmed the association between 321 differentially expressed probes between *DOCK1*-high and *DOCK1*-low patients (Supplementary Table 10) (with *t*-test p<0.05 and >1.5-fold change between *DOCK1*-high and -low patients) and functions involved in chemotaxis and cell migration-related functions, cell proliferation, and apoptosis (modified Fisher's exact test p-values<0.05; Figure 3B). Overall, our data implied the involvement of *DOCK1* in chemotaxis and migration in hematopoietic cells, at least partially accounting for unfavorable survival in AML patients with higher *DOCK1* expression. Further biological investigations are needed to delineate the underlying mechanisms.

## DISCUSSION

While DOCK proteins have pleiotropic functions in immunity, neurology, cytokinesis, and motility, their functions in hematopoietic system and prognostic significance in AML are totally unknown. In this study, we addressed these questions by analyzing the expression patterns of the 11 *DOCK* member genes in normal hematopoietic and AML cells. The data were extracted from ours and other public databases. Our analyses focused on *DOCK1* since it was the only one in the family with prognostic significance. The finding can be validated in other independent cohorts.

DOCK1 protein is involved in the regulation of actin cytoskeleton and cell motility [2]. The dysregulation of these molecules may be responsible for invasive and metastatic properties of cancer cells [9, 10, 19]. *DOCK1* gene expression carries prognostic significance in breast cancer, ovarian cancer and glioblastoma multiforme through activation of c-JUN, STAT3, and Rac1 [9, 10, 19]. Given these findings in solid cancers, the role of DOCK1 protein in AML remains unexplored. To the best of our knowledge, the current study is the first to report the clinical implication of *DOCK* gene expression in AML. *DOCK1* gene seems to be a special *DOCK* member because of its unique enrichment in HSC and adverse prognostic impact in AML.

The mechanisms underlying poor prognostic implication of higher *DOCK1* expression in AML remain to be explored. Our bioinformatics approach revealed *DOCK1* overexpression coincided with some of its known functions such as cell migration, motion, and chemotaxis in AML cells (Supplementary Table 10). HIF-1α [20, 21] and CXCR4, two factors related to cell motion and chemotaxis, have been reported as unfavorable prognostic markers for AML [22, 23]. The analysis of microarray data exhibited significant correlation of the expression of *DOCK1* with that of *HIF-1α* and *CXCR4* in our cohort (data not shown). This was consistent with previous study in which knock down of *ELMO1*, the partner gene of *DOCK1*, resulted in reduced chemotaxis of leukemia cells [3].

We showed that *DOCK1* was associated with both HSC and LSC signatures. Since stemness is an established property pertaining to drug resistance and poor prognosis in cancer patients [24], these data provided an explanation for the unfavorable prognostic impact of *DOCK1* over-expression in AML. We compared the prognostic significance of *DOCK1* expression to several published multi-gene signatures originally derived from a wide range of molecular mechanisms (*e.g*., epigenetic changes and chemoresistance genes) and cohorts with different cytogenetic subtypes and races. The comparisons were performed among datasets from both cytogenetically normal (GSE12417) and cytogenetically heterogeneous AML patients (NTUH and TCGA) (Table 3). The promising performance of *DOCK1* expression reinforces its potential as a simple and powerful prognostic marker that seems to be independent of cytogenetic abnormalities, but further studies are necessary to confirm it.

We noted that the three datasets analyzed in this study were derived from different profiling platforms. Probe-wise z-transformation was thus performed to each dataset in order to eliminate cross-platform and cross-cohort biases. For simplicity, in this study we adopted a median of *DOCK1* expression as a cutoff level. For clinical practice, a receiver operating characteristic (ROC) analysis of 2-year survival in the NTUH dataset suggested an optimal cutoff at 0.625 (z-transformed expression of *DOCK1*, or, if multiple probes exist, average of z-transformed expression of all probes of *DOCK1*). The cutoff achieved significant differences in survival curves in the three datasets (all p-values<0.001) and warrants further testing by a large prospective study.

In summary, our study concluded that the overexpression of *DOCK1* as an unfavorable prognostic marker in acute myeloid leukemia possibly through its correlation with stemness, cell proliferation, motility and chemotaxis. Current study focused on the clinical and bioinformatics analyses on the prognostic and biological impacts of *DOCK1* in AML. Prospective studies are warranted to confirm our observation. Moreover, further mechanistic studies are necessary to delineate how *DOCK1* participates in the stem cell biology of hematopoietic lineages and its role in modulating unfavorable prognostic impact in AML.

## MATERIALS AND METHODS

### Patients

We recruited 347 adult patients with newly diagnosed AML at the National Taiwan University Hospital (NTUH) from 1995 to 2011. Among them, 227 patients underwent standard intensive chemotherapy as described previously [25]. We obtained written informed consent from each patient in accordance with Declaration of Helsinki. This study was approved by the Research Ethics Committee of the National Taiwan University Hospital.

### Cytogenetic and mutation analyses

Cytogenetic risk was defined according to the European Leukemia Net (ELN) guideline [26]. Analyses of genetic mutations including *FTL3*-ITD*, FTL3*-TKD, *NPM1, MLL*-PTD*, NRAS, KRAS, PTPN11, KIT, JAK2, WTI, CEBPA, RUNX1, ASXL1, IDH1, IDH2, TET2* and *DNTM3A* mutations were carried out in bone marrow mononuclear cells obtained at diagnosis as described previously [27–31].

### High-throughput mRNA assay and data analysis

Illumina HumanHT-12 v4 Expression BeadChips (Illumina, Inc., San Diego, CA) were utilized to profile global gene expression of bone marrow cells from the 347 AML patients as previously described (GEO accession numbers: GSE68469 and GSE71014) [14, 32, 33]. Expression of *DOCK1* was analyzed in binomial manner with a median cutoff to ensure balance comparison, as in previous literatures [13, 15, 32]. To confirm the prognostic implication of *DOCK1* in our cohort, we incorporated publicly available gene expression microarray datasets from two independent AML cohorts, The Cancer Genome Atlas (TCGA; n=186) [34] and GSE12417 (n=162) [35]. The RNA-Seq dataset of the TCGA cohort (n=179) was also utilized in this study which was represented in RPKM (reads per kilobase of transcript per million mapped reads) counts. All microarray data were converted to the log-2 scale if not performed originally. Probe expression levels were transformed to *z*-values (subtraction of probe mean and division by probe standard deviation) and averaged into gene-level data.

### Functional enrichment analysis

Differentially expressed genes associated with the gene of interest (*i.e*., *DOCK1*) were analyzed by the Database for Annotation, Visualization and Integrated Discovery (DAVID) [36, 37] for related functions, such as Gene Ontology terms and biological pathways, with default settings. We also utilized the Gene Set Enrichment Analysis (GSEA) [38] to investigate the association between expression profile of the gene and other biological functions or gene signatures. Briefly, previously published or our manually curated gene signatures were collected and tested for enrichment in a genome-wide gene list, which was sorted by the significance of differential expression between patients with highest (>3^rd^ quartile) and lowest (<1^st^ quartile) expression of the gene. We adopted default GSEA settings and the significance of an enrichment score was evaluated by a permutation test with respect to genes.

### Statistical analysis

Statistical analysis was generated using SPSS software 21.0. We first examine the normality of array parameter with Shapiro-Wilk test. Non-parametric variables were analyzed with Mann-Whitney test while nominal parameters were compared by Chi-square test. The overall survival (OS) was calculated and plotted with the use of Kaplan-Meier curves from the date of first diagnosis to date of last follow-up or death from any cause. The disease free survival (DFS) was measured from the date of primary treatment to the date of disease relapse or death from any cause. Log-rank test was utilized to determine statistical significance. Whole patient cohort (n=347) was included in the analysis of association between *DOCK* expression levels and clinical characteristics yet only the 227 of them who received standard chemotherapy were included for survival analyses. The prognostic significance of parameters such as age, white blood cell counts, CD34 expression, mutation status of the afore-mentioned 16 genes and expression levels of *DOCK1* were first evaluated individually in univariate analysis and the significant factors were then examined together with multivariate analysis using Cox regression. Level of significance was set at p<0.05.

## SUPPLEMENTARY FIGURE AND TABLES




