# Supplementary material for: High expression of dedicator of cytokinesis 1 (DOCK1) confers poor prognosis in acute myeloid leukemia
Source: Oncotarget. 2017 Jul 31;8(42):72250–9. doi: 10.18632/oncotarget.19706 (PMC5641127; doi:10.18632/oncotarget.19706)
Supplement: Supplementary file 1 [file oncotarget-08-72250-s001.pdf]

High expression of dedicator of cytokinesis 1 (DOCK1) confers poor prognosis in acute myeloid leukemia

SUPPLEMENTARY MATERIALS

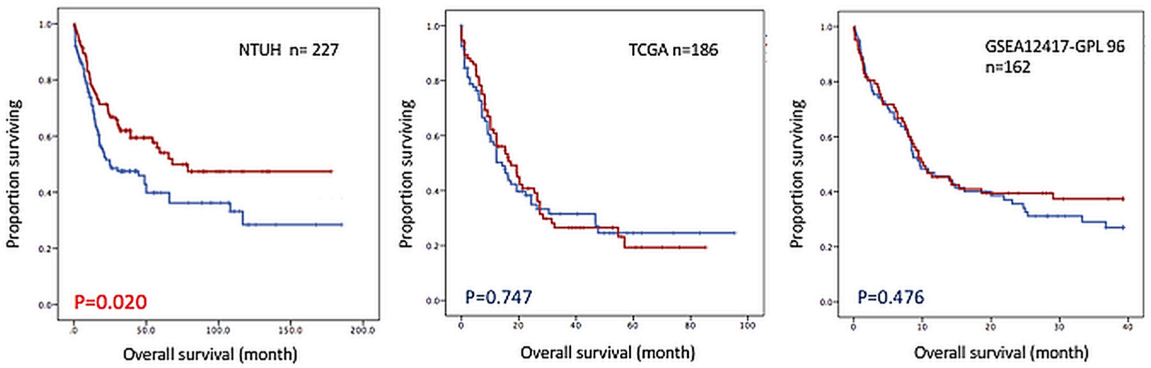

Supplementary Figure 1: Kaplan Meier survival curves for AML patients stratified by *DOCK2* expression levels. Overall survival of AML patients with different *DOCK2* levels in all the three independent cohorts (NTUH, TCGA and GSEA12417-GPL 96). Red line: higher *DOCK1* expression; blue line: lower *DOCK1* expression.

Supplementary Table 1: Univariate analysis on the overall survival in the AML patients

See Supplememtary File 1

Supplementary Table 2: Univariate analysis on the disease free survival in the AML patients

See Supplememtary File 1

Supplementary Table 3: Multivariate analysis (Cox regression) on the disease free survival*

Variables	Diease free survival			
	HR	95% CI		*P* value
		Lower	Upper	
**Total cohort (n=227)**				
Age	1.013	1.001	1.025	0.033
WBC	1.000	1.000	1.000	0.142
Unfavorable cytogenetics	2.426	1.390	4.235	0.002
FLT3-ITD	1.155	0.751	1.777	0.512
CEBPA$^{double\ mutation}$	0.765	0.390	1.501	0.436
RUNX1 mutation	1.399	0.826	2.370	0.212
DNMT3A mutation	1.350	0.875	2.083	0.175
MLL-PTD	1.372	0.624	3.014	0.432
TP53 mutation	2.237	0.832	6.015	0.111
***DOCK1* higher expression**	**1.659**	**1.262**	**2.182**	**<0.001**

*The model was generated from a stepwise Cox regression model that included age, WBC, unfavorable cytogenetics, gene mutations of *FLT3*, *CEBPA*, *RUNX1*, DNTM3A, *MLL*, *TP53* and expression level of *DOCK1*.

HR, hazard ratio; CI, confidence interval; WBC, white blood cell count.

Supplementary Table 4: Association of DOCK1 expression levels with cytogenetic abnormalities

Variables	Total	Higher *DOCK1* expression	Lower *DOCK1* expression	*P*
**Karyotype †**	347	174	173	<0.001
Favorable	60	8 (13.3%)	52 (86.7%)	<0.001
Intermediate	223	125 (56.1%)	98 (43.9%)	0.003
Unfavorable	49	29 (59.2%)	20 (40.8%)	0.172
Unknown	15	12 (80%)	3 (20%)	0.018
Normal	167	92 (55.1%)	75 (44.9%)	0.021
t(8;21)	24	1 (4.2%)	23 (95.8%)	<0.001
t(15;17)	27	1 (3.7%)	26 (96.3%)	<0.001
inv(16)	9	6 (66.7%)	3 (33.3%)	0.277

†Favorable, t(15;17), t(8;21), inv (16) or t(16;16); unfavorable, inv(3), t(3;3), t(6;9), t(v;11)(v;q23), -7, -5, del(5q), abnl(17p), complex abnormalities‡; Intermediate, t(9;11), normal karyotype and other abnormalities not classified as favorable or adverse.

‡ Complex karyotype is defined as three or more chromosome abnormalitiesin the absence of one of the WHO designated recurring translocations or inversions: t(8;21), inv(16) or t(16;16), t(15;17), t(9;11), t(v;11)(v;q23), t(6;9), inv(3) or t(3;3).

Supplementary Table 5: Association of BM DOCK1 expression level with other genetic alterations

Variables	No. of patients with alteration (%)			*P*
	Whole cohort (n=347)	Higher DOCK1 expression (n=174)	Lower DOCK1 expression (n=173)	
FLT3/ITD	84	57 (67.9%)	27 (32.1%)	<0.001
FLT3/TKD	32	19 (59.4%)	13 (40.6%)	0.273
N-RAS	59	28 (47.5%)	31 (52.5%)	0.651
K-RAS	15	6 (40%)	9 (60%)	0.422
PTPN11	22	16 (72.7%)	6 (27.3%)	0.029
KIT	15	3 (20%)	12 (80%)	0.017
MLL	13	11 (84.6%)	2 (15.4%)	0.024
WTI	26	17 (65.4%)	9 (34.6%)	0.106
NPM1	99	67 (67.7%)	32 (32.3%)	<0.001
CEBPA	27	4 (14.8%)	23 (85.2%)	<0.001
RUNX1	50	34 (68%)	16 (32%)	0.006
TP53	16	10 (62.5%)	6 (37.5%)	0.317
ASXL1	52	40 (76.9%)	12 (23.1%)	<0.001
IDH1	20	11 (55%)	9 (45%)	0.655
IDH2	51	19 (37.3%)	32 (62.7%)	0.046
TET2	56	31 (55.4%)	25 (44.6%)	0.394
DNMT3A	66	41 (62.1%)	25 (37.9%)	0.031

Supplementary Table 6: Leading-edge genes of HSC gene set

Gene symbol	Rank in genome-wide list	Metric score (-log P)	Up-regulation	Running ES
MEIS1	3	16.788	High *DOCK1*	0.0600
KIAA0125	4	16.067	High *DOCK1*	0.1176
HOXB2	5	15.628	High *DOCK1*	0.1735
SPINK2	7	15.231	High *DOCK1*	0.2280
HOXA5	11	13.915	High *DOCK1*	0.2778
HOXB3	21	11.87	High *DOCK1*	0.3200
DAPK1	67	8.239	High *DOCK1*	0.3482
CD109	87	7.45	High *DOCK1*	0.3743
WBP5	95	7.231	High *DOCK1*	0.4000
LPP	104	6.909	High *DOCK1*	0.4245
WDR91	162	5.781	High *DOCK1*	0.4436
FNBP1	164	5.757	High *DOCK1*	0.4642
FLT3	250	4.72	High *DOCK1*	0.4786
ZEB1	289	4.361	High *DOCK1*	0.4931
SOCS2	290	4.351	High *DOCK1*	0.5087
GUCY1A3	312	4.215	High *DOCK1*	0.5232
MYO5C	330	4.136	High *DOCK1*	0.5375
PRKCH	341	4.093	High *DOCK1*	0.5519
KBTBD8	342	4.092	High *DOCK1*	0.5665
KLF4	426	3.723	High *DOCK1*	0.5774
MSI2	469	3.52	High *DOCK1*	0.5888
HTR1F	477	3.49	High *DOCK1*	0.6011
TPT1	480	3.477	High *DOCK1*	0.6135
TMEM38B	633	3.021	High *DOCK1*	0.6199
HLF	652	2.993	High *DOCK1*	0.6301
TMEM200A	706	2.878	High *DOCK1*	0.6389
ZDHHC21	850	2.651	High *DOCK1*	0.6442
DUSP6	1054	2.361	High *DOCK1*	0.6468
ATP8B4	1073	2.339	High *DOCK1*	0.6546
RIMKLB	1127	2.278	High *DOCK1*	0.6612
COL5A1	1300	2.113	High *DOCK1*	0.6638
YES1	1535	1.912	High *DOCK1*	0.6639
CRIM1	1669	1.819	High *DOCK1*	0.6665

Supplementary Table 7: Leading-edge genes of LSC gene set

Gene symbol	Rank in genome-wide list	Metric score (-log P)	Up-regulation	Running ES
RABGAP1	111	6.783	High *DOCK1*	0.1392
NAB1	346	4.081	High *DOCK1*	0.2180
SLC9A7	439	3.666	High *DOCK1*	0.2923
ATP1B1	473	3.501	High *DOCK1*	0.3648
CLN5	1146	2.256	High *DOCK1*	0.3927
PNPLA4	1294	2.121	High *DOCK1*	0.4330
EIF2S3	1420	2.014	High *DOCK1*	0.4716
PPP1R10	1993	1.617	High *DOCK1*	0.4890
ZFP30	2370	1.438	High *DOCK1*	0.5083
ARL3	2455	1.402	High *DOCK1*	0.5353
PTCD2	2923	1.214	High *DOCK1*	0.5473
CRKRS	3488	1.043	High *DOCK1*	0.5528
FLJ13197	3592	1.012	High *DOCK1*	0.5710
ZNF500	4678	0.753	High *DOCK1*	0.5554
MAP3K7	4693	0.749	High *DOCK1*	0.5707
LRRC8B	4880	0.713	High *DOCK1*	0.5803
PPIG	5336	0.631	High *DOCK1*	0.5804

Supplementary Table 8: Leading-edge genes of Homeobox gene set

Gene symbol	Rank in genome-wide list	Metric score (-log P)	Up-regulation	Running ES
HOXB2	5	15.628	High *DOCK1*	0.1093
HOXA5	11	13.915	High *DOCK1*	0.2066
HOXB5	14	12.835	High *DOCK1*	0.2965
HOXB3	21	11.87	High *DOCK1*	0.3794
HOXB4	27	11.039	High *DOCK1*	0.4566
HOXB7	33	10.285	High *DOCK1*	0.5285
HOXA7	42	9.439	High *DOCK1*	0.5944
HOXA6	46	9.267	High *DOCK1*	0.6592
HOXB8	57	8.717	High *DOCK1*	0.7200
HOXA9	188	5.453	High *DOCK1*	0.7544
HOXA2	220	5.003	High *DOCK1*	0.7886
HOXA11	266	4.571	High *DOCK1*	0.8193
HOXB9	432	3.688	High *DOCK1*	0.8403
HOXA10	536	3.285	High *DOCK1*	0.8604

Supplementary Table 9: Leading-edge genes of ELMO1-interaction gene set

Gene symbol	Rank in genome-wide list	Metric score (-log P)	Up-regulation	Running ES
DOCK1	0	45.626	High *DOCK1*	0.4398
GNGT1	612	3.062	High *DOCK1*	0.4517
KLF3	678	2.937	High *DOCK1*	0.4781
RDX	908	2.545	High *DOCK1*	0.4960
CLIP3	1273	2.139	High *DOCK1*	0.5061
TTC19	1602	1.863	High *DOCK1*	0.5145
MSN	1828	1.714	High *DOCK1*	0.5245

Supplementary Table 10: Differentially-expressed probes between DOCK1-high and DOCK1-low patients

See Supplememtary File 1
