# Supplementary material for: High expression of dedicator of cytokinesis 1 (DOCK1) confers poor prognosis in acute myeloid leukemia
Source: Oncotarget. 2017 Jul 31;8(42):72250–9. doi: 10.18632/oncotarget.19706 (PMC5641127; doi:10.18632/oncotarget.19706)
Supplement: Supplementary file 2 [file oncotarget-08-72250-s002.docx]

**Supplementary Table 1: Univariate analysis on the overall survival in the AML patients**

| Variable^‡^ | **Overall survival** | |
| --- | --- | --- |
|  | Months^#^ | P |
| **Age** |  | 0.003 |
| >=50 | 108.1 (59.5- 156.7) |  |
| <50 | 23.4 (0.613-46.2) |  |
| **WBC** |  | 0.015 |
| >=20000 | 30.1 (10.4- 49.8) |  |
| <20000 | 116.8 |  |
| **Karyotype** |  | <0.001 |
| Unfavorable | 13.2 (9.6-16.8) |  |
| Otherwise | 66.0 (23.8-108.2) |  |
| ***NPM1*** |  | 0.639 |
| Mutated | 50.0(40.0-60.0) |  |
| Wild | 54.4(34.7-74.1) |  |
| ***FLT3-*ITD*^+^*** |  | 0.001 |
| Mutated | 18.0(12.3-23.7) |  |
| Wild | 108.1(82.5-133.7) |  |
| ***NPM1+/ FLT3-ITD^-^*** |  | 0.210 |
| Yes | 116.8 (41.6-191.9) |  |
| Others | 44.7 (22.5-66.9) |  |
| ***FLT3-TKD*** |  | 0.828 |
| Mutated | 39.2(12.3-66.1) |  |
| Wild | 50.0(38.8-61.2) |  |
| ***CEBPA*** |  | 0.002 |
| Double mutation | NR |  |
| Others | 39.2(28.9-49.5) |  |
| ***RUNX1*** |  | 0.040 |
| Mutated | 24.9(17.0-32.7) |  |
| Wild | 59.3(37.5-81.0) |  |
| ***WT1*** |  | 0.020 |
| Mutated | 14.7(12.4-17.0) |  |
| Wild | 59.3(47.8-70.9) |  |
| ***IDH2*** |  | 0.430 |
| Mutated | 66.0(41.4-90.6) |  |
| Wild | 50.0(37.9-62.1) |  |
| ***ASXL1*** |  | 0.815 |
| Mutated | 22.0 |  |
| Wild | 54.4(43.2-65.6) |  |
| ***DNMT3A*** |  | 0.313 |
| Mutated | 39.2(22.8-55.6) |  |
| Wild | 59.3(46.2-72.4) |  |
| ***IDH1*** |  | 0.958 |
| Mutated | 57.4(24.4-90.4) |  |
| Wild | 50.0(38.4-61.6) |  |
| ***TET2*** |  | 0.093 |
| Mutated | 16.0(9.4-22.6) |  |
| Wild | 57.4(48.9-65.9) |  |
| ***PTPN11*** |  | 0.898 |
| Mutated | NR |  |
| Wild | 50.0(38.3-61.7) |  |
| ***NRAS*** |  | 0.160 |
| Mutated | 66.0 |  |
| Wild | 48.8(37.7-59.9) |  |
| ***KRAS*** |  | 0.164 |
| Mutated | 14.0(0.1-27.9) |  |
| Wild | 54.4(44.9-63.9) |  |
| ***MLL-*PTD** |  | 0.001 |
| Mutated | 10.5(6.3-14.7) |  |
| Wild | 57.4(47.25-67.55) |  |
| ***TP53*** |  | <0.001 |
| Mutated | 2.5(1.3-3.7) |  |
| Wild | 57.4(47.9-66.9) |  |
| **KIT** |  | 0.350 |
| Mutated | 17.4(10.5-24.3) |  |
| Wild | 54.4(42.2-66.4) |  |
| ***CD34*** |  | 0.018 |
| Presence | 31.1 (11.8-50.4) |  |
| Absence | 116.8 |  |
| ***DOCK1*** |  | <0.001 |
| Higher expression | 18.0 (10.1-25.9) |  |
| Lower expression | 116.8 |  |

NR, not reached

^#^Months: median± 95% CI

**Supplementary Table 2: Univariate analysis on the disease free survival in the AML patients**

| Variable^‡^ | **Disease free survival** | |
| --- | --- | --- |
|  | Months^#^ | P |
| **Age** |  | 0.009 |
| >=50 | 9.4(4.5-14.3) |  |
| <50 | 21.0 (0-75.3) |  |
| **WBC** |  | 0.027 |
| >=20000 | 9.4 (6.7-12.1) |  |
| <20000 | 27.0 (0-87.4) |  |
| **Karyotype** |  | 0.001 |
| Unfavorable | 0 |  |
| Otherwise | 15(6.4-23.7) |  |
| ***NPM1*** |  | 0.997 |
| Mutated | 17.5(12.4-22.6) |  |
| Wild | 11.0(5.8-16.2) |  |
| ***FLT3-*ITD*^+^*** |  | 0.030 |
| Mutated | 7.5(3.2-11.8) |  |
| Wild | 18.1(6.3-30.0) |  |
| ***NPM1+/ FLT3-ITD^-^*** |  | 0.131 |
| Yes | 45.8 |  |
| Others | 11.0(7.0-15.0) |  |
| ***FLT3-TKD*** |  | 0.785 |
| Mutated | 18.1(7.7-28.5) |  |
| Wild | 13.0(8.2-17.8) |  |
| ***CEBPA*** |  | 0.037 |
| Double mutation | NR |  |
| Others | 11.0(6.8-15.2) |  |
| ***RUNX1*** |  | 0.013 |
| Mutated | 5.6(0.8-10.4) |  |
| Wild | 15.0(9.0-20.9) |  |
| ***WT1*** |  | 0.111 |
| Mutated | 7.0(3.7-10.3) |  |
| Wild | 15.0(9.3-20.7) |  |
| ***IDH2*** |  | 0.652 |
| Mutated | 13.0(3.0-23.0) |  |
| Wild | 14.0(8.7-19.3) |  |
| ***ASXL1*** |  | 0.433 |
| Mutated | 19.7 |  |
| Wild | 13.0(8.1-17.9) |  |
| ***DNMT3A*** |  | 0.020 |
| Mutated | 7.0(4.4-9.6) |  |
| Wild | 15.5(5.9-25.1) |  |
| ***IDH1*** |  | 0.505 |
| Mutated | 15.5(0-33.0) |  |
| Wild | 13.0(6.9-19.1) |  |
| ***TET2*** |  | 0.419 |
| Mutated | 9.0(4.2-13.8) |  |
| Wild | 15.0(9.3-20.7) |  |
| ***PTPN11*** |  | 0.910 |
| Mutated | 19.0 |  |
| Wild | 13.0(7.8-18.2) |  |
| ***NRAS*** |  | 0.844 |
| Mutated | 15.0(2.1-27.9) |  |
| Wild | 12.5(7.0-18.0) |  |
| ***KRAS*** |  | 0.103 |
| Mutated | 5.8(0-18.2) |  |
| Wild | 14.5(8.8-20.2) |  |
| ***MLL-*PTD** |  | 0.004 |
| Mutated | 2.5(0-9.8) |  |
| Wild | 15.0(9.3-20.7) |  |
| ***TP53*** |  | <0.001 |
| Mutated | 0 |  |
| Wild | 14.5(9.0-20.0) |  |
| **KIT** |  | 0.329 |
| Mutated | 7.7(6.5-8.9) |  |
| Wild | 14.5(8.8-20.2) |  |
| ***CD34*** |  | 0.003 |
| Presence | 9.0 (6.6-11.39) |  |
| Absence | 45.8 |  |
| ***DOCK1*** |  | <0.001 |
| Higher expression | 6.4(4.3-8.5) |  |
| Lower expression | 96.4 |  |

NR, not reached

^#^Months: median±95% CI

**Supplementary Table 10: Differentially-expressed probes between DOCK1-high and DOCK1-low patients**

| **Probe** | **Gene symbol** | ***T*-test *P*-value** | **Log-2 ratio** |
| --- | --- | --- | --- |
| **2360196** | *DOCK1* | 2.37E-46 | 1.49 |
| **630465** | *SCHIP1* | 4.66E-23 | 1.76 |
| **7330435** | *CCDC6* | 1.62E-17 | 0.73 |
| **4040753** | *MEIS1* | 1.63E-17 | 1.35 |
| **3780647** | *KIAA0125* | 8.56E-17 | 1.74 |
| **3120475** | *MAP7* | 2.25E-16 | 1.3 |
| **3460097** | *HOXB2* | 2.36E-16 | 2.02 |
| **2810673** | *HOXA9* | 2.81E-16 | 2.76 |
| **4290431** | *DPYSL3* | 2.93E-16 | 1.91 |
| **1030484** | *SPINK2* | 5.87E-16 | 2.41 |
| **3780619** | *TMEM14A* | 1.75E-15 | 0.86 |
| **6330187** | *PLS1* | 2.05E-15 | 0.61 |
| **5820491** | *MAP7* | 2.41E-15 | 1.26 |
| **510520** | *ARHGAP22* | 1.16E-14 | 1.65 |
| **6620437** | *HOXA5* | 1.21E-14 | 2.98 |
| **510097** | *HOXA10* | 1.26E-14 | 2.23 |
| **2680110** | *TM4SF1* | 4.71E-14 | 1.95 |
| **4280136** | *ADRM1* | 1.32E-13 | -0.75 |
| **1470500** | *HOXB5* | 1.46E-13 | 2.17 |
| **60553** | *NDFIP1* | 2.94E-13 | 0.88 |
| **6420050** | *NPTX2* | 3.03E-13 | 1.68 |
| **5340646** | *ASAP2* | 4.17E-13 | 0.65 |
| **380390** | *FAM30A* | 5.07E-13 | 1.39 |
| **4540184** | *SYTL4* | 5.43E-13 | 0.86 |
| **580592** | *CPNE8* | 9.43E-13 | 1.14 |
| **2350575** | *HOXB3* | 1.35E-12 | 1.12 |
| **520465** | *LCT* | 1.48E-12 | 0.76 |
| **3400240** | *PRDM16* | 1.62E-12 | 1.09 |
| **4230746** | *TRPC2* | 1.64E-12 | 0.61 |
| **6660195** | *RPUSD3* | 2.15E-12 | -0.74 |
| **5560561** | *TNS3* | 2.16E-12 | 1.46 |
| **1470386** | *CPNE8* | 3.40E-12 | 1.54 |
| **5910689** | *SMC4* | 6.87E-12 | 0.96 |
| **2120008** | *HOXB4* | 9.14E-12 | 0.76 |
| **4390121** | *ADRM1* | 1.32E-11 | -0.62 |
| **4280678** | *SEPP1* | 1.89E-11 | 1.63 |
| **1340026** | *TMEM144* | 1.90E-11 | 0.67 |
| **2030041** | *DDEF2* | 2.23E-11 | 0.6 |
| **3990553** | *HS.576106* | 4.26E-11 | 0.9 |
| **2470328** | *HOXB7* | 5.19E-11 | 1.13 |
| **1400446** | *PLIN2* | 7.98E-11 | -0.9 |
| **2900427** | *P2RX5* | 8.17E-11 | -0.82 |
| **1690561** | *ARPP-21* | 9.61E-11 | -0.78 |
| **1110615** | *BEX4* | 1.01E-10 | 1.19 |
| **4480170** | *LOC729086* | 1.14E-10 | 0.95 |
| **7330538** | *PYHIN1* | 1.15E-10 | -0.74 |
| **160382** | *TRH* | 1.44E-10 | -1.91 |
| **7040682** | *HS.25318* | 1.74E-10 | 1.13 |
| **4560301** | *ARPP-21* | 1.88E-10 | -0.81 |
| **1470551** | *SEL1L3* | 1.95E-10 | 1.18 |
| **4120114** | *IL12A* | 2.21E-10 | 0.73 |
| **5360246** | *NKX2-3* | 2.24E-10 | 1.34 |
| **7160553** | *HOXA4* | 2.41E-10 | 0.72 |
| **2760500** | *CD38* | 2.89E-10 | -0.82 |
| **6650348** | *LAPTM4B* | 3.03E-10 | 1.36 |
| **580132** | *LAPTM4B* | 3.63E-10 | 1.44 |
| **6020494** | *LOC404266* | 3.68E-10 | 1.7 |
| **2510672** | *TCF4* | 3.76E-10 | 0.84 |
| **4730309** | *PCCA* | 4.73E-10 | 0.87 |
| **3140537** | *LRRC28* | 5.34E-10 | -0.89 |
| **610563** | *HOXA6* | 5.41E-10 | 1.37 |
| **7510408** | *NRIP1* | 6.06E-10 | 0.84 |
| **2900048** | *HOXA3* | 6.92E-10 | 1.07 |
| **1470427** | *ALDH4A1* | 9.10E-10 | -0.71 |
| **3130451** | *HGF* | 1.30E-09 | -1.31 |
| **5490768** | *GPR56* | 1.36E-09 | 1.36 |
| **3930326** | *LOC728014* | 1.50E-09 | -0.66 |
| **2640224** | *VEGFA* | 1.75E-09 | -0.66 |
| **870524** | *HOXB8* | 1.92E-09 | 1.77 |
| **1510019** | *MGST2* | 1.98E-09 | 0.7 |
| **4640446** | *FHL1* | 2.03E-09 | 0.81 |
| **6220189** | *HOXB6* | 2.41E-09 | 0.94 |
| **3140603** | *SVIL* | 2.57E-09 | 0.71 |
| **7550301** | *SLC8A3* | 2.59E-09 | 0.66 |
| **4220605** | *ETS2* | 2.74E-09 | 0.77 |
| **6590709** | *RHPN1* | 2.76E-09 | 0.66 |
| **3390021** | *SDSL* | 3.08E-09 | 0.91 |
| **380019** | *DDIT4L* | 3.30E-09 | -0.94 |
| **6250435** | *ARHGEF3* | 3.30E-09 | 0.83 |
| **3400538** | *IGFBP7* | 3.47E-09 | -0.93 |
| **2340292** | *HGF* | 3.75E-09 | -0.9 |
| **4890241** | *GPR56* | 5.43E-09 | 1.29 |
| **5700220** | *DAPK1* | 5.77E-09 | 0.66 |
| **5870138** | *VWF* | 9.81E-09 | 1.18 |
| **4050452** | *PPA1* | 1.13E-08 | 0.72 |
| **7000270** | *PYHIN1* | 1.15E-08 | -0.82 |
| **2480274** | *SORL1* | 1.25E-08 | -0.81 |
| **6200670** | *LPIN1* | 1.30E-08 | 0.73 |
| **460204** | *PLIN2* | 1.63E-08 | -0.88 |
| **5420592** | *SRC* | 1.66E-08 | 0.72 |
| **730458** | *LRP3* | 1.69E-08 | -1.05 |
| **4560743** | *CD96* | 1.72E-08 | -1.53 |
| **2470634** | *PBX3* | 2.07E-08 | 1.28 |
| **6940360** | *ACVR1* | 2.09E-08 | 0.68 |
| **1070477** | *ALDH1A1* | 2.94E-08 | 1.01 |
| **2710754** | *CD96* | 3.02E-08 | -1.59 |
| **6560546** | *ITPKA* | 3.15E-08 | -0.71 |
| **4670059** | *ZAK* | 3.66E-08 | 0.94 |
| **2900041** | *H2AFY2* | 3.89E-08 | 1.21 |
| **6290114** | *SDPR* | 3.99E-08 | 0.83 |
| **840253** | *ALDH2* | 4.31E-08 | 1.13 |
| **3870324** | *CPVL* | 4.37E-08 | 1.38 |
| **5340162** | *KIAA1598* | 5.32E-08 | 1.1 |
| **1660750** | *WBP5* | 5.52E-08 | 0.66 |
| **7510731** | *WBP5* | 5.88E-08 | 0.95 |
| **60136** | *CPVL* | 6.39E-08 | 1.54 |
| **2710039** | *RGS10* | 6.72E-08 | -0.59 |
| **5890121** | *NRG4* | 8.30E-08 | 0.75 |
| **5860717** | *ROBO3* | 1.07E-07 | 0.79 |
| **7550048** | *CERCAM* | 1.46E-07 | -0.72 |
| **2450246** | *OSBPL5* | 1.46E-07 | -0.63 |
| **7320382** | *SLC38A1* | 1.50E-07 | 0.99 |
| **3520343** | *C10ORF140* | 1.52E-07 | 1.09 |
| **4890674** | *SH3BP4* | 1.92E-07 | 0.76 |
| **6270301** | *RAB33A* | 2.17E-07 | -0.66 |
| **3170440** | *BCL11A* | 2.77E-07 | 0.72 |
| **5080601** | *COBLL1* | 2.88E-07 | 0.72 |
| **2450639** | *TSC22D1* | 2.96E-07 | 0.91 |
| **4490180** | *BEXL1* | 3.14E-07 | 1.07 |
| **6290161** | *SLC16A9* | 3.44E-07 | 0.71 |
| **150647** | *NTNG2* | 3.46E-07 | -0.61 |
| **380689** | *TSC22D1* | 3.90E-07 | 0.87 |
| **2970594** | *MYADM* | 4.25E-07 | 0.6 |
| **2510356** | *EMR1* | 4.77E-07 | 0.73 |
| **1300546** | *VSTM1* | 5.29E-07 | -1.06 |
| **2350279** | *TMEM44* | 6.76E-07 | -0.64 |
| **7400598** | *POU4F1* | 8.12E-07 | -1.55 |
| **5690398** | *TNFSF4* | 8.40E-07 | 0.61 |
| **6590735** | *NYNRIN* | 8.82E-07 | 0.84 |
| **70026** | *SYNGR1* | 9.26E-07 | -0.67 |
| **5670142** | *LOC100132535* | 9.53E-07 | -0.61 |
| **7400368** | *RUNX1* | 9.82E-07 | 0.61 |
| **160148** | *AK3L1* | 1.02E-06 | -0.61 |
| **6510754** | *ALDH1A1* | 1.02E-06 | 0.77 |
| **4570575** | *ST7* | 1.03E-06 | -0.6 |
| **7570215** | *CPNE3* | 1.07E-06 | 0.8 |
| **4060692** | *SUCLG2* | 1.24E-06 | 0.79 |
| **6580270** | *LOC646723* | 1.31E-06 | 1.19 |
| **2680202** | *HYAL3* | 1.32E-06 | -0.77 |
| **2450427** | *NKG7* | 1.35E-06 | -0.67 |
| **7560541** | *SLC2A5* | 1.37E-06 | 0.93 |
| **1260543** | *ARHGAP21* | 1.40E-06 | 0.89 |
| **5260288** | *DUSP6* | 1.42E-06 | 0.75 |
| **6100180** | *EFEMP2* | 1.59E-06 | -0.7 |
| **1240474** | *ZBTB16* | 1.66E-06 | -0.67 |
| **1410097** | *KRT8P9* | 1.76E-06 | 0.58 |
| **7320193** | *GHRL* | 1.83E-06 | -0.61 |
| **1030204** | *CCL1* | 1.92E-06 | 0.79 |
| **5340201** | *LOC100132535* | 2.00E-06 | -0.85 |
| **5860722** | *MIR155HG* | 2.03E-06 | 0.66 |
| **5720129** | *NFIX* | 2.29E-06 | 0.76 |
| **5910594** | *PTP4A3* | 2.35E-06 | 0.75 |
| **5690477** | *LOC729645* | 2.52E-06 | -1.75 |
| **1260228** | *CLEC5A* | 2.77E-06 | -0.99 |
| **4230735** | *FAM129A* | 2.91E-06 | -0.67 |
| **4880333** | *FLJ40504* | 3.01E-06 | 0.91 |
| **1070482** | *HPGDS* | 3.24E-06 | -0.63 |
| **4180338** | *LGALS12* | 3.29E-06 | -0.6 |
| **450615** | *MT2A* | 3.75E-06 | -0.9 |
| **2680605** | *IGJ* | 3.87E-06 | 0.93 |
| **2810059** | *KLF4* | 4.27E-06 | 1.08 |
| **1300470** | *MAMDC2* | 4.42E-06 | 1.29 |
| **6770474** | *CYP2S1* | 4.92E-06 | 0.74 |
| **5080450** | *ZBTB16* | 5.08E-06 | -0.68 |
| **5260349** | *NGFRAP1* | 5.54E-06 | 0.67 |
| **4920040** | *DEFB1* | 5.92E-06 | 1.6 |
| **6620392** | *LFNG* | 7.21E-06 | 0.89 |
| **4290037** | *NOV* | 7.74E-06 | 0.79 |
| **7650524** | *BCAT1* | 8.07E-06 | 0.74 |
| **2640544** | *ASS1* | 8.50E-06 | -0.68 |
| **2060477** | *AGPAT9* | 8.61E-06 | 0.68 |
| **3440491** | *GALC* | 1.10E-05 | -0.6 |
| **670731** | *CYTL1* | 1.15E-05 | -1.32 |
| **670348** | *GPA33* | 1.32E-05 | -0.62 |
| **6860162** | *LOC441019* | 1.33E-05 | -0.68 |
| **1050309** | *HLX* | 1.34E-05 | 0.73 |
| **1690692** | *SOCS2* | 1.43E-05 | 0.84 |
| **6560136** | *CAST* | 1.47E-05 | 0.66 |
| **3290044** | *TTC27* | 1.52E-05 | 0.61 |
| **460113** | *MAGED1* | 1.56E-05 | 0.81 |
| **840324** | *CLDN10* | 1.59E-05 | 0.86 |
| **3370768** | *C1ORF24* | 1.60E-05 | -0.6 |
| **270706** | *COL4A5* | 1.60E-05 | 1.09 |
| **6180095** | *LOC730029* | 1.61E-05 | 0.62 |
| **3800600** | *MAGED1* | 1.62E-05 | 0.71 |
| **3360066** | *ANGPT1* | 1.63E-05 | 0.83 |
| **3850246** | *HOPX* | 1.63E-05 | 1.27 |
| **7560433** | *DSG2* | 1.72E-05 | 0.62 |
| **4860681** | *GUCY1A3* | 1.73E-05 | 0.96 |
| **4610753** | *ENG* | 1.74E-05 | 0.67 |
| **3870102** | *HNMT* | 1.77E-05 | 0.7 |
| **2850451** | *FLT3* | 1.91E-05 | 0.64 |
| **3460053** | *SIPA1L2* | 2.05E-05 | -0.76 |
| **6940039** | *SORL1* | 2.10E-05 | -0.66 |
| **510332** | *HS.505676* | 2.26E-05 | -0.7 |
| **6370435** | *ETS1* | 2.32E-05 | 0.67 |
| **5690671** | *GOLGA8B* | 2.43E-05 | 0.88 |
| **5570768** | *HOXA11AS* | 2.56E-05 | 0.84 |
| **3990017** | *RUNX1T1* | 2.59E-05 | -0.59 |
| **5260754** | *TSC22D1* | 2.82E-05 | 0.83 |
| **3120326** | *ANGPT1* | 2.93E-05 | 0.94 |
| **2350441** | *COL23A1* | 2.99E-05 | -0.7 |
| **5310327** | *HS.390407* | 3.37E-05 | -0.61 |
| **7040369** | *MSI2* | 3.44E-05 | 0.69 |
| **6110709** | *LOC284998* | 3.66E-05 | -0.7 |
| **3870301** | *SORBS3* | 3.75E-05 | 0.61 |
| **5720136** | *RAB34* | 3.81E-05 | 0.74 |
| **2140121** | *BASP1* | 3.87E-05 | 1.18 |
| **6770673** | *SOCS2* | 4.46E-05 | 1 |
| **6510554** | *DACH1* | 4.74E-05 | 0.64 |
| **2760079** | *FOXC1* | 4.79E-05 | 1.54 |
| **1710189** | *RHBDF1* | 4.91E-05 | 0.77 |
| **4280327** | *C2ORF82* | 5.15E-05 | -0.76 |
| **4220468** | *ATP1B1* | 5.43E-05 | 0.65 |
| **6590026** | *ZNF521* | 5.53E-05 | 0.83 |
| **4230678** | *HIST2H2BE* | 6.90E-05 | 0.69 |
| **2320296** | *KCNK17* | 7.40E-05 | 0.68 |
| **3290731** | *PRKCH* | 8.07E-05 | 0.61 |
| **2710121** | *LOC442597* | 8.17E-05 | -0.85 |
| **450553** | *AHNAK* | 8.43E-05 | 0.6 |
| **2260468** | *KCNE1L* | 9.04E-05 | -1.18 |
| **6860220** | *NGFRAP1* | 9.55E-05 | 0.96 |
| **4730368** | *LOC390183* | 9.55E-05 | 0.62 |
| **4490520** | *EBI2* | 0.00011152 | 0.82 |
| **110433** | *ASS1* | 0.00011325 | -0.8 |
| **2450519** | *LOC645630* | 0.00012197 | 0.64 |
| **4210382** | *DLL3* | 0.00012451 | -0.65 |
| **6040008** | *SKAP2* | 0.00013302 | 0.61 |
| **3840767** | *TSPAN7* | 0.00013623 | -0.72 |
| **1050008** | *MMRN1* | 0.00013845 | 0.59 |
| **20129** | *CD52* | 0.00014356 | 0.86 |
| **1580411** | *CD3D* | 0.00015022 | -0.76 |
| **7570484** | *TFF3* | 0.00015286 | -0.8 |
| **2140541** | *LOC389816* | 0.00016933 | 0.66 |
| **3520370** | *MSI2* | 0.00017814 | 0.6 |
| **2030403** | *OLIG1* | 0.00018052 | -0.69 |
| **7000577** | *GYPC* | 0.00018408 | -0.71 |
| **7210136** | *CD19* | 0.00018527 | -0.7 |
| **510044** | *KLF4* | 0.00018905 | 0.71 |
| **4830674** | *BEX2* | 0.0002053 | 0.6 |
| **2230241** | *F13A1* | 0.00021279 | 0.89 |
| **6650242** | *IFITM3* | 0.00023882 | 1.13 |
| **3710202** | *MST1* | 0.00026102 | -0.6 |
| **5420646** | *CETP* | 0.00026287 | -0.59 |
| **2070520** | *CDCA7* | 0.00026534 | -0.72 |
| **3780674** | *ZNF503* | 0.00026547 | 0.66 |
| **7330215** | *SVOPL* | 0.00028587 | -0.73 |
| **6330487** | *SULF2* | 0.00030981 | 0.63 |
| **2320653** | *ECHDC2* | 0.00031866 | 0.72 |
| **7200242** | *SULF2* | 0.00038403 | 0.67 |
| **7380273** | *GPR114* | 0.00044094 | 0.71 |
| **2030142** | *CLC* | 0.00046528 | 1.18 |
| **2600187** | *LOC728843* | 0.00047327 | 0.63 |
| **4810520** | *TRIB1* | 0.00048641 | 0.72 |
| **6280440** | *BIK* | 0.00053485 | 0.67 |
| **5360156** | *IFITM1* | 0.00056439 | -0.71 |
| **4810341** | *CST7* | 0.00060227 | -0.77 |
| **1070278** | *LOC643308* | 0.00061046 | 0.62 |
| **2710709** | *FCGR1B* | 0.00061092 | -0.64 |
| **5050681** | *TESC* | 0.0006192 | -0.65 |
| **4610129** | *RETN* | 0.00070632 | -0.98 |
| **2900129** | *FTO* | 0.00072342 | 0.64 |
| **7210398** | *GYPC* | 0.00074893 | -0.66 |
| **3800403** | *APOC2* | 0.0007921 | -0.61 |
| **1170671** | *CD3D* | 0.00081439 | -0.66 |
| **1170709** | *CDH2* | 0.00088008 | 0.77 |
| **5960682** | *RBPMS2* | 0.0008981 | -0.85 |
| **1400711** | *ANXA8* | 0.00092957 | -0.61 |
| **6200402** | *MT1A* | 0.00094917 | -0.63 |
| **4040037** | *EBI2* | 0.00097029 | 0.59 |
| **460010** | *TSPAN7* | 0.0010218 | -0.81 |
| **5820551** | *CT45A4* | 0.0010833 | 0.93 |
| **2810156** | *IGF2R* | 0.0011342 | 0.7 |
| **4540239** | *DEFA1* | 0.0011573 | 0.76 |
| **1410021** | *AIF1L* | 0.0011684 | 0.84 |
| **1580465** | *AMICA1* | 0.0013598 | 0.69 |
| **6940358** | *SELL* | 0.0014534 | 0.68 |
| **2230563** | *PPBP* | 0.0014703 | 0.85 |
| **7000465** | *RGL4* | 0.0014802 | -0.73 |
| **5490408** | *CEBPD* | 0.0017874 | -0.67 |
| **1230603** | *CT45A4* | 0.0018204 | 0.8 |
| **3290259** | *NAPSB* | 0.0018763 | 0.99 |
| **6620292** | *C10ORF54* | 0.0019313 | 0.59 |
| **4860128** | *DEFA1B* | 0.0019744 | 0.86 |
| **2970747** | *DEFA3* | 0.0022311 | 0.85 |
| **4040176** | *LAMA5* | 0.0022852 | -0.78 |
| **7150170** | *DEFA1B* | 0.0029191 | 0.93 |
| **1580576** | *ITM2A* | 0.0030137 | -0.71 |
| **1940047** | *AIF1* | 0.0034841 | 0.63 |
| **240086** | *PHGDH* | 0.0036958 | 0.72 |
| **5360477** | *LOC643332* | 0.0037386 | 0.7 |
| **870477** | *DEFA1B* | 0.0038166 | 0.89 |
| **2450400** | *RASGRP3* | 0.0040173 | 0.61 |
| **2030767** | *CD48* | 0.0040576 | -0.63 |
| **2100296** | *NAPSA* | 0.004086 | 0.59 |
| **1510424** | *S100P* | 0.0044844 | -0.89 |
| **1170300** | *MT1G* | 0.0046398 | -0.66 |
| **7650064** | *UMODL1* | 0.0046467 | -0.63 |
| **2140707** | *SLPI* | 0.0049595 | -0.67 |
| **770400** | *LOC653600* | 0.0051804 | 0.99 |
| **60184** | *NDN* | 0.0062324 | 0.68 |
| **4010040** | *HBG2* | 0.0070798 | -0.97 |
| **7510128** | *CT45A4* | 0.0071172 | 0.63 |
| **7400113** | *HBG1* | 0.0078706 | -0.95 |
| **4850301** | *PTRF* | 0.0079137 | 0.74 |
| **3310538** | *CD36* | 0.007962 | 0.71 |
| **2810010** | *CCL3L3* | 0.0082739 | 0.72 |
| **2070168** | *CX3CR1* | 0.0094253 | 0.7 |
| **6280243** | *DNTT* | 0.010293 | 0.83 |
| **4540682** | *PALM* | 0.010529 | -0.61 |
| **1010592** | *CD36* | 0.011463 | 0.65 |
| **430204** | *MYCN* | 0.011732 | 0.77 |
| **6280326** | *HDC* | 0.013242 | -0.75 |
| **3360113** | *MEG3* | 0.014467 | -0.63 |
| **7200520** | *DNTT* | 0.017067 | 0.67 |
| **6280646** | *LOC100133662* | 0.022294 | -0.86 |
| **6590682** | *CCL3* | 0.024232 | 0.61 |
| **5690408** | *IRX3* | 0.024571 | 0.87 |
| **2340743** | *CPA3* | 0.024848 | 0.74 |
| **6100687** | *RPS4Y1* | 0.025846 | -0.92 |
| **4150600** | *EIF1AY* | 0.030819 | -0.72 |
